# Design of a Phased Array EMAT for Inspection Applications in Liquid Sodium

**DOI:** 10.3390/s19204460

**Published:** 2019-10-15

**Authors:** Laura Pucci, Raphaële Raillon, Laura Taupin, François Baqué

**Affiliations:** 1 French Alternative Energies and Nuclear Energy Commission—Laboratory for Integration of Systems and Technology (CEA-LIST) – Digiteo Labs, 91191 Gif-sur-Yvette Cedex, France; laura.pucci@cea.fr (L.P.); raphaele.raillon@cea.fr (R.R.); 2 French Alternative Energies and Nuclear Energy Commission—Division of Nuclear Energy (CEA-DEN) – 13108 Saint-Paul-lez-Durance cedex, France; francois.baque@cea.fr

**Keywords:** EMAT sensor, Phased Array, L-waves, liquid sodium, inspection, NDE, SFR

## Abstract

This article describes the development of a French CEA in-house phased array Electro Magnetic Acoustic Transducer (EMAT) adapted to hot and opaque sodium environment for in-service inspection of Sodium Fast Reactors. The work presented herein aimed at improving in-service inspection techniques for the ASTRID reactor project. The design process of the phased array EMAT is explained and followed by a review of laboratory experimental test results.

## 1. Introduction

In-service inspection of Sodium Fast Reactors (SFR) requires the development of non-destructive techniques adapted to the hot (about 200°C) and opaque sodium environment. Piezoelectric probes are usually used for in-service inspection; however, in liquid sodium, this kind of probes encounters wetting problems. The liquid sodium does not adhere to the surface of the probe, leading to the presence of gas at the probe’s surface that impedes an effective transmission of the ultrasounds in the liquid sodium. To deal with this issue, piezoelectric probes have to be immerged several hours in liquid sodium at very high temperature, which leads to damaging risks for these probes. Electro Magnetic Acoustic Transducers (EMAT) have shown promise for overcoming these wetting problems, as their technology enables the generation of pressure waves directly inside the liquid sodium, and therefore, using EMATs simplifies and accelerates the implementation of inspections in such environment. The R&D work concerning non-destructive inspection techniques for the ASTRID reactor (see [1] for an overview of the current status of the associated R&D program) has therefore aimed at exploring the potential of the EMAT technique, especially concerning under-sodium viewing and in situ distance measurements in the reactor pressure vessel. In that context, other ultrasonic transducers, based on piezoelectric concept, are being developed for Non-Destructive Examination (TUCSS of FRAMATOME [2]) and for Under Sodium Vision (IMARSOD of CEA [3]).

Previous work performed at the CEA-LIST Non-Destructive Testing Department has led to proofs of concept and liquid sodium feasibility tests using single element EMATs, as well as phased array EMATs [4,5,6]. These probes happened not to be sensitive enough, and the recent work has been focused on the ways to overcome this lack of sensitivity. This eventually led to a 12-element phased array EMAT sensitive enough to consider trials in liquid sodium.

In this paper, the development process of a 12-element optimized phased array EMAT will be first described, then, testing results obtained in the laboratory on aluminum blocks with this probe will be presented.

## 2. Context

Since France in 2008 considered the SFR concept to be the most mature for Generation IV nuclear reactors, an extensive R&D program was launched. In-Service Inspection was identified as a difficult task to perform (as sodium coolant is opaque, hot, and highly chemically reactive, as well as being difficult to drain). Ultrasonic techniques have been extensively studied, as they are well adapted to Non-Destructive Examination and telemetry measurement in this harsh environment.

Thus, development of ultrasonic transducers to be immersed in sodium at about 200 °C in the reactor block (inspection is performed at shutdown conditions) led to a first phase of specification consolidation, followed by a pre-qualification process involving increasingly more realistic experiments using acoustic techniques and simulations performed with the patented CIVA code [7].

Associated applications for inspection deal with telemetry, vision and volumetric control for SFR reactor block systems, structures and components, and also for the power conversion system. EMAT concept appears as an efficient candidate for alternative technology of piezoelectric ultrasonic sensors.

**Figure 1:**
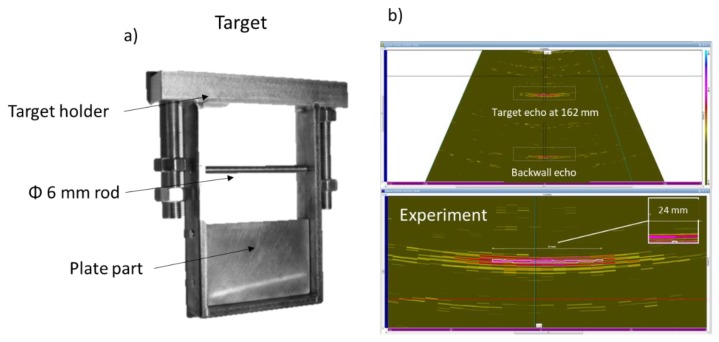
(**a**): Picture of the target used for liquid sodium test with the previous EMAT. (**b**): Ultrasonic scanning images during liquid sodium testing.

Previous work has led to an 8-element phased array EMAT, tested under liquid sodium environment at 180 °C for at least 24 h [6]. The EMAT was located 160 mm from the target, as shown in Figure 1. The target presents two parts: the first one is composed of a flat plate, and the second of a 6-mm-diameter rod. The flat plate is used as a reference, as all the energy sent to this part comes back to the sensor if its surface is parallel to the plate. Thus, this reference is used to adjust the orientation of the probe relative to the target. Then, the second part of the target is used to verify the angular beam deflection of the EMAT and its resolution on a rod. An angular scan with several positions was performed. The obtained image is shown in Figure 1. An echo is observed after the plate echo; this comes from the container wall behind the target. 

The obtained image suffers from a high noise level, which does not allow discrimination of the finer details of the advanced target (the 6 mm tube, for instance). The aim of the work described in this paper is to improve the sensitivity of the EMAT in order to be able to detect this kind of detail. However, these results with low sensitivity still demonstrate the proper functioning, over several hours, of the EMAT in a harsh environment (liquid sodium at 180 °C). 

## 3. Development of the Optimized 12-Element Phased Array EMAT

**Figure 2:**
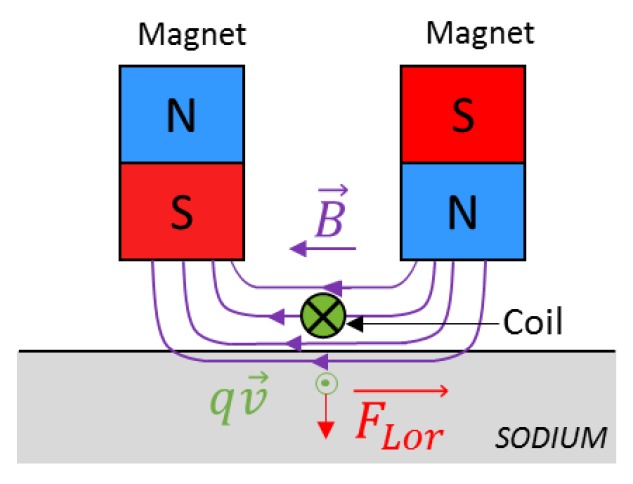
Physical principles of pressure wave generation in liquid sodium. $q\vec{v}$ is the electric current density, $\vec{B}$ the magnetic field, $\vec{F_{Lor}}$ the Lorentz force.

The principle of EMAT force generation in the context of under sodium viewing is explained in Figure 2. A static magnetic field is induced in the region of the liquid sodium by permanent magnets. In liquid, only Longitudinal-waves (L-waves) can be generated, in which case the orientation of the static magnetic field delivered by permanent magnets is suitable for generating such ultrasonic waves. In addition to this field, eddy currents are generated directly inside the liquid sodium thanks to the coil, which is located between the two magnets, powered by an alternative current. The penetration depth of the eddy currents depends on the material conductivity and permeability and the pulse frequency of the coil alimentation. This is called skin depth. In liquid sodium, for a pulse frequency of 1 MHz, the skin depth is approximatively 110 µm. The cross product between the eddy current distribution and the static magnetic field results in a pulsed volume Lorentz force, which is located in the skin depth, and acts as a source for pressure waves. This force can be expressed by the following equation: F→Lor=q.v→^B→ (Figure 2). These ultrasonic pressure waves can then be used for inspection purposes in the liquid sodium. Potential applications include object detection, distance measurements, surface metrology, imaging for exploratory purposes and non-destructing testing of components. The design shown in Figure 2 supports the generation of ultrasonic vertical L-waves. 

Based on this principle, a phased array EMAT was developed. The aim is to find the most suitable arrangement for the magnets and the coils for generating L-waves that are able to focus or deflect the L-beam and reach a sufficient sensitivity. This last point requires precisely considering the electronic aspect, which is essential for this kind of probe, the low transduction efficiency of which is well known.

### 3.1. Magnet and Coil Design

The phased array EMAT is made up of several coils. Each coil represents an element to which a delay can be applied in order to focalize or deflect the L ultrasonic beam by applying the appropriate delay laws to all the elements.

The challenge for this EMAT is to combine the need for a small pitch (the pitch is the distance between the centers of two adjacent elements (coils)) to ensure constructive interferences of the ultrasonic waves, and the need for large magnets and coils to ensure a sufficient sensitivity. Indeed, in order to be able to deflect the ultrasonic beam without increasing the grating lobes, it is recommended to fix the pitch “p” so that p/λ is lower than 1/2. With “λ” being the wavelength of the L-waves in liquid sodium that satisfies the formula λ = c/f, where “c” is the celerity of the L-waves in liquid sodium, and “f” is the center frequency of the EMAT excitation signal. For the targeted frequencies (1 to 2 MHz), this constraint imposes a pitch lower than 1.23 to 0.6 mm.

**Figure 3:**
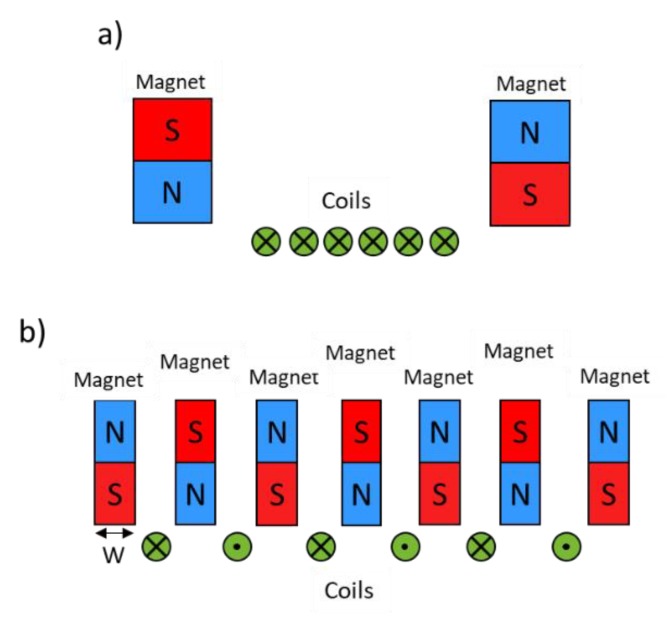
Diagram of two possible designs of the EMAT probe.

Two designs were considered for the phased array EMAT probe; they are represented in Figure 3.

In diagram a), two strong magnets surround all of the elements (coils). This is the design of the first EMAT built for this project. Its main advantages are a small pitch and a strong magnetic field, which is achieved thanks to the large magnets. The main disadvantages are due to the large distance between the two magnets. This leads to an inhomogeneous magnetic field over the elements: the amplitude of the Lorentz forces generated by each coil decreases rapidly with distance from the magnet, preventing a good mastery of the beam. It also limits the number of elements. Because of these limitations, the other arrangement (b)) for the EMAT was chosen.

In diagram b), each element (coil) is surrounded by two magnets, allowing the same magnetic field over each element. Moreover, there is no more issue concerning the increase of the number of elements. However, the size of the magnets (width, “W” in Figure 3, is limited here, because the larger their size, the larger the pitch, and the greater the amplitude of the grating lobes. Furthermore, small magnets lead to weak magnetic fields, and therefore to weak probe sensitivity. Therefore, a compromise should be found between the pitch value and the magnets size. Please note that the same considerations remain valid for the coil width, as it impacts the pitch. Moreover, another consideration has to be taken into account regarding the coil width. Indeed, the EMAT is planned to operate in both emission and reception. In emission, a large coil width is needed to allow the coil to be crossed by high currents that will generate intensive ultrasonic waves, but in reception, a narrow coil width is needed to increase the sensitivity. These two antagonistic requirements impose a trade-off with respect to the coil width. 

It can be noticed that two coils next to each other are supplied by current in opposite directions. This is due to the fact that the direction of the static magnetic field above one coil is in a direction opposite to that above the other. Then, in order to obtain constructive Lorentz forces, the current flowing through two adjacent coils needs to be in opposite directions.

Some simulations were performed with CIVA software in order to define the coil and magnet sizes for a 1 MHz excitation signal in liquid sodium. The results led to the following values: the magnets were 3 mm wide, 25 mm in height and 23 mm in length, and the coils width was 1.05 mm. The resulting pitch was 4.05 mm. 

This value of the pitch, 4.05 mm, is significantly superior to the recommended value. The consequence is the presence of important grating lobes as displayed later. 

### 3.2. Increase of the Magnetic Field

**Figure 4:**
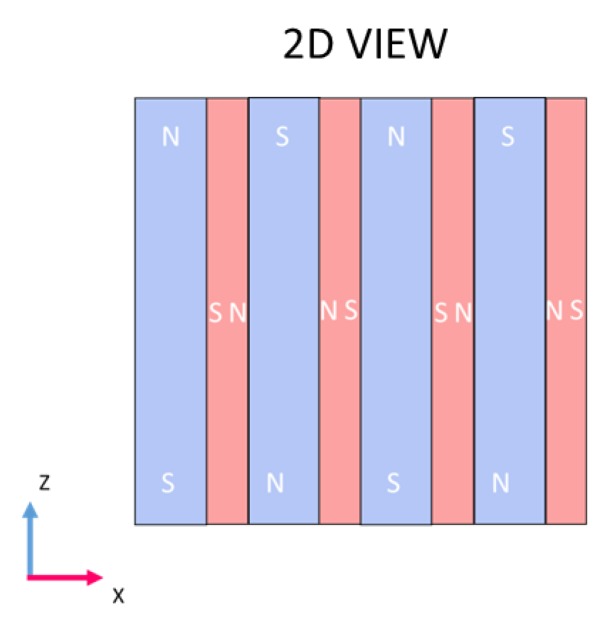
Magnetic assembly design of the EMAT probe with the “Klaus Halbach” design.

Once the dimensions of the coils and the magnets were chosen, the increase of the magnetic field was studied. Magnets magnetized along the $\vec{X}$ axis were interleaved between the magnets magnetized along the $\vec{Z}$ axis (Figure 4**) **in order to increase the magnetic field along the $\vec{X}$ axis at the surface of the sodium. This kind of design is called a “Halbach array” [8], and is depicted in Figure 4.

**Figure 5:**
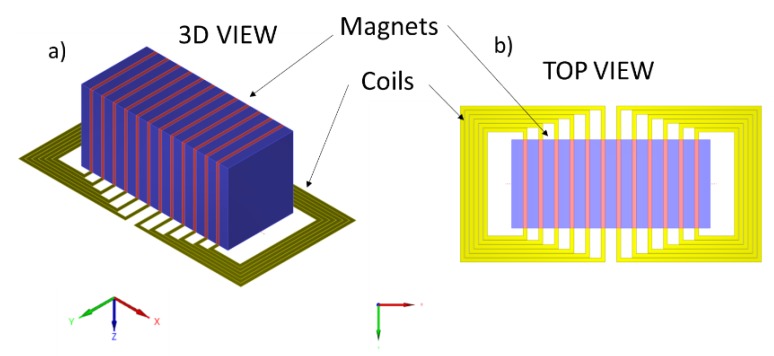
(**a**): 3D view of the EMAT. (**b**): top view of the EMAT.

In Figure 5, diagrams of the EMAT are exposed. The twelve rectangular coils are etched on a flexible PCB and made of 8 turns. Each coil represents one element. 

This new magnet arrangement increases the magnetic field close to the surface (0.1 mm) by about a factor of 2 and by a factor of 1.5 at higher distances (1 mm).

**Figure 6:**
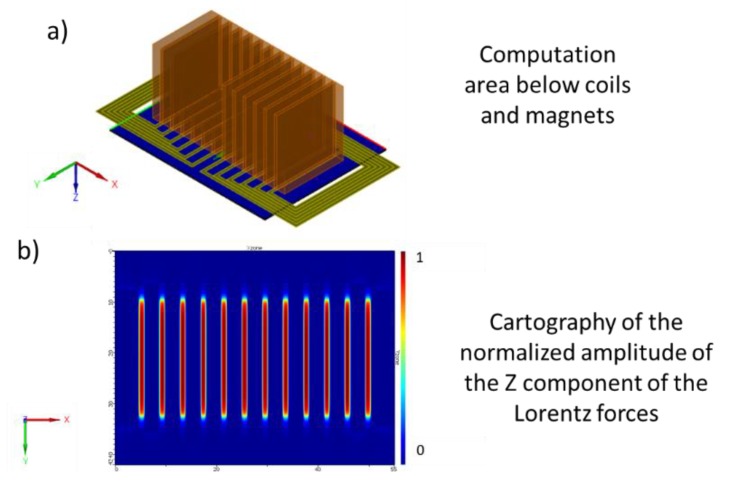
Simulation result. Normalized Z component of the Lorentz forces of the 12-element EMAT in liquid sodium. (**a**): Computation area below the sensor. (**b**): Cartography of normalized amplitude of the Z component of the Lorentz forces.

The cartography of the amplitude of the vertical component of the Lorentz force generated below the 12-element EMAT was computed with CIVA in liquid sodium (Figure 6). As can be seen, the amplitude of the vertical components of the Lorentz force distribution are the same for each element, as required.

The magnets used in this study (laboratory experiments) were Neodymium-Iron-Boron magnets, because they are well known for delivering a strong magnetic field at room temperature. However, their magnetizing force decreases with temperature increase until they lose their magnetism at around 590 °C. Magnets with a higher Curie point should be used for measurements in liquid sodium at 180 °C. For the sodium trials, we used Samarium-Cobalt magnets with a Curie point at around 1000 °C.

### 3.3. Resonance Phenomena

**Figure 7:**
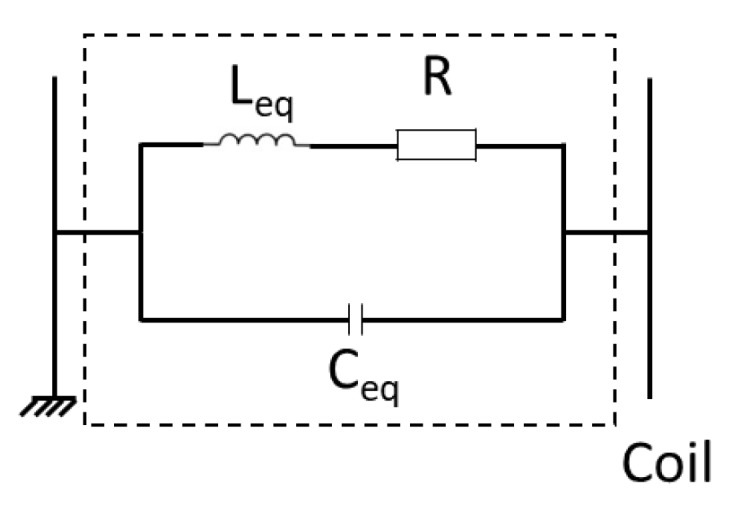
Equivalent electronic model proposed by Jian et al. [9] for one element of the EMAT.

After the choice of the geometric parameters of the EMAT, the electronic aspect was studied. The equivalent electronic model proposed by Jian et al. [9] for one coil is shown in Figure 7.

This electronic circuit presents a resonance frequency described by Thomson formula, where L_eq_ is the equivalent inductance of the coil and C_eq_ is the equivalent capacity of the coil.

$$f=\;\frac{1}{\sqrt{2\pi L_{eq}C_{eq}}}$$

**Figure 8:**
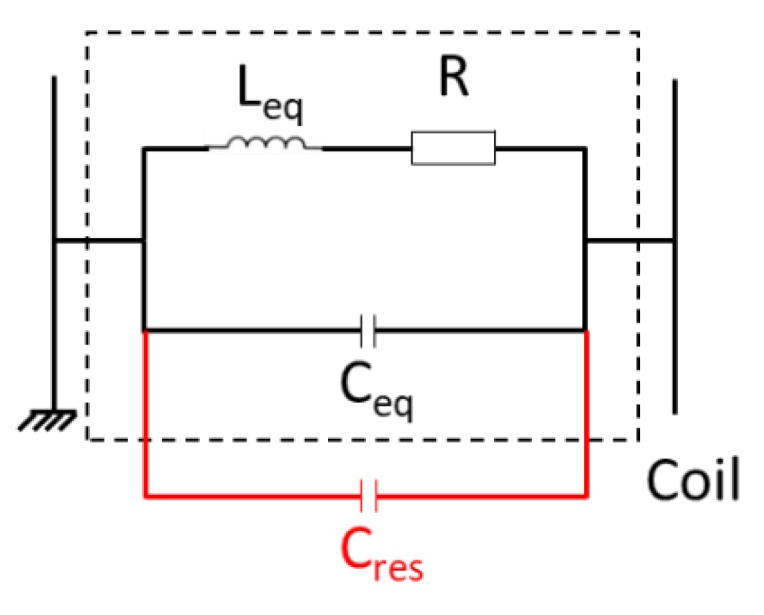
Equivalent model of a coil with the addition of a capacitor in order to operate at the resonance frequency.

To improve the amplitude of the emitted and received signals of the EMAT, the resonance frequency formula must be verified for each coil at the center frequency “f” of the EMAT excitation signal. To adjust the parameters of the coil (L_eq_ and C_eq_) to the work frequency (f) the value of the capacitor can be modified by adding a parallel capacitor, named C_res_, as represented in Figure 8. The determination of the value of C_res_ is made theoretically for each coil by resolving the Thomson formula (knowing f and L_eq_). The theoretical value is then adjusted experimentally by a current measurement in each coil. 

In this second section, the geometry of the phase array EMAT and the method for optimizing the sensitivity of the probe (magnet arrangement and use of the resonance phenomena) have been described.

## 4. Characteristics of The Ultrasonic Beam in Liquid Sodium with Civa Simulation Software

In this third part, the acoustic beam characteristics of the EMAT were checked thanks to the CIVA software. The beam radiated by the 12-element phased array EMAT in liquid sodium at 180 °C was computed with CIVA. Different delay laws were applied to the EMAT in order to check its capacity to focus and deflect the beam.

### 4.1. Focusing without Angular Deflection

**Figure 9:**
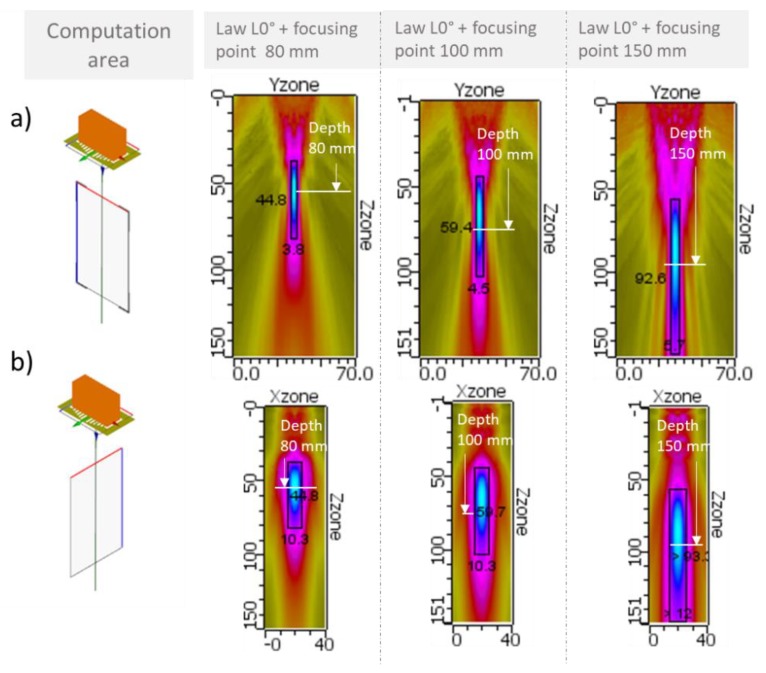
Simulation results. Longitudinal ultrasonic field radiated in liquid sodium by the 12-element EMAT. Delay laws applied to focus at 80 mm, 100 mm and 150 mm without beam deflection (law L0°). Computation of the field in two 2D areas located in the focal plane (figures a)) and in the orthogonal plane (figures b)). Excitation signal at 1 MHz composed of 3 bursts (signal close to the experimental one used in Section 4).

A first set of delay laws was applied in order to focus the L-waves of the ultrasonic beam at depths of 80, 100 and 150 mm without angular deflection (delay law called “L0°”). The results of the simulated beams are shown in Figure 9. The cartographies displayed on this figure show the maximum amplitude of the temporal acoustic signal radiated at each point of the cartography. The color blue corresponds to the maximum amplitude and the color green to the smallest amplitude.

In the focal plane (images a) in Figure 9), the focal length varied from 45 mm to 93 mm when the focusing depth increased. The focal distances required in the 3 cases were reached. In the case of a focusing point at 150 mm, the presence of grating lobes very close to the main beam can be noticed. As it is not recommended to work with such an ultrasonic beam, a shorter focusing distance will be chosen.

In the orthogonal plane (images b) in Figure 9), the focal width varied from 10.3 to 12 mm when the focusing depth increased. 

For each of those three simulations, the amplitude of the ultrasonic beam displayed is normalized. Nevertheless, a comparison of the three maximum radiated amplitudes was carried out. The simulation results show that the maximal amplitude of the beam decreased with the increase of the focal depth point. There was a loss of 3 dB between 80 mm and 150 mm focusing depths. 

Considering the ultrasonic beam profile and amplitude, a focusing point at 100 mm was chosen for the next simulation step. 

### 4.2. Focusing with Angular Deflection

**Figure 10:**
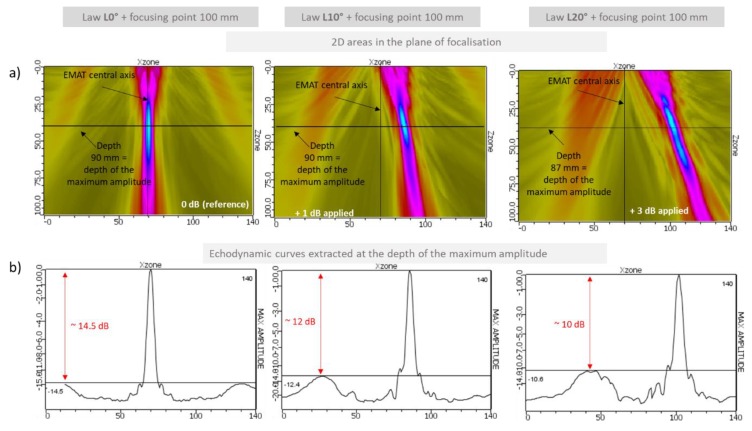
Simulation results. Longitudinal ultrasonic field radiated in 180 °C liquid sodium by the 12-element EMAT. Focusing law at 100 mm without deflection (left figure), with 10° deflection (middle figures) and with 20° deflection (right figure). Computation of the field in a 2D area located in the focal plane (figures a)) and amplitude of the field along a horizontal profile extracted from the 2D field ate the depth of the maximal amplitude (figures b)). Excitation signal at 1 MHz composed of 3 bursts (signal close to the experimental one used in Section 4).

A second set of delay laws was applied in order to focus and deflect the L-wave beam. The focal distance chosen was 100 mm and the deflection angles were 0°, 10° and 20°. The simulated beams are shown in Figure 10.

These maps show that the beam was well deflected. However, as expected, because of the large value of the pitch with respect to the operating frequency, the amplitude of the grating lobes was significant and increased with the required deflection angle. Thus, it will be important to ensure that they do not lead to erroneous interpretation of the echoes, and simulations will be helpful for that.

The simulation results shown in Section 2 and Section 3 validate the parameters chosen for the EMAT. However, CIVA does not predict EMAT sensitivity. The EMAT sensitivity was then evaluated on the basis of experimental trials performed on aluminum blocks.

## 5. Validation of Probe Performances Under Laboratory Conditions

In this section, the experimental results obtained with the EMAT on aluminum blocks are reported. The frequency used for these experimental measurements was 2 MHz, in order to obtain about the same wavelength in aluminum at this frequency (3.2 mm) as in liquid sodium at 1 MHz (2.5 mm). 

### 5.1. Transmission Measurement on Aluminum Block

**Figure 11:**
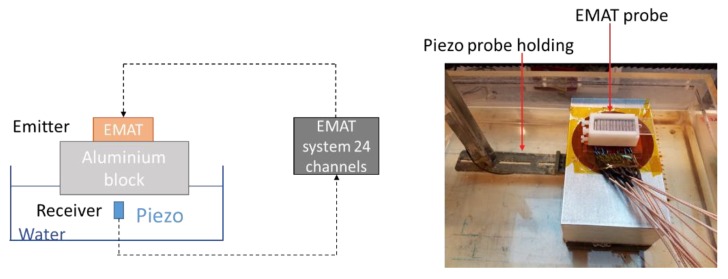
Experimental setup for transmission measurement—emission by the EMAT and reception by the piezoelectric probe.

A first objective was to characterize the radiated beams obtained with different delay laws. Therefore, an 80**-**mm-thick aluminum block was used in a transmission configuration, with the EMAT and a fixed emitter located on one side of the block, and a piezoelectric probe and receiver scanning the surface on the other side of the block. This configuration is shown in Figure 11. 

**Figure 12:**
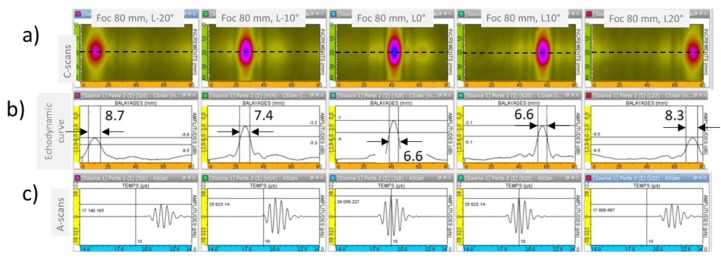
Experimental results obtained for five delay laws applied to focus the L beam at 80 mm depth with angular deflections of L-20°, L-10°, L0°, L10° and L20° (from left to right). (**a**): C-scans; (**b**): Echodynamic curves; and (**c**): A-scans extracted at the position of the maximum amplitude of the C-scans.

Five delay laws were applied for focusing the longitudinal wave beam radiated at the backwall of the block (80 mm) with different angles of deflection (−20°, −10°, 0°, 10° and 20°). An alternative voltage of 250 V at 2 MHz composed of 3 bursts supplied the coils of the EMAT. The signals measured by the piezoelectric transducer placed close to the backwall of the aluminum block are presented in Figure 12. The C-scans displayed represent the maximum amplitude of the signal recorded with the piezoelectric probe at each position of the scan.

It can be noticed that the ultrasonic beams were deflected as expected, with a good spatial resolution. The focal width at −3 dB does not increase much with the deflection, as can be seen on the echodynamic curves (6.6 mm for L0° and 8.7 mm for L20°). The maximal amplitude radiated at 80 mm decreases with deflection: there is a loss of 4.6 dB between the L0° and the L20° deflections.

In addition, the amplitude of the grating lobes observed around the main beam are approximatively 12 dB weaker than the maximal amplitude at the focal point.

### 5.2. Pulse Echo Measurement on Aluminum Block

**Figure 13:**
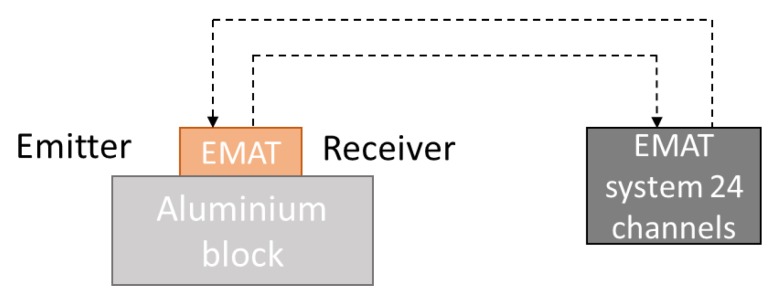
Experimental setup for pulse echo measurement—emission and reception by the EMAT.

In this section, the results obtained with the EMAT probe in pulse echo mode are exposed. The aluminum block used is the same as the one described in Section 4.1. The alimentation of the coils also remained identical. This configuration is shown in Figure 13. 

**Figure 14:**
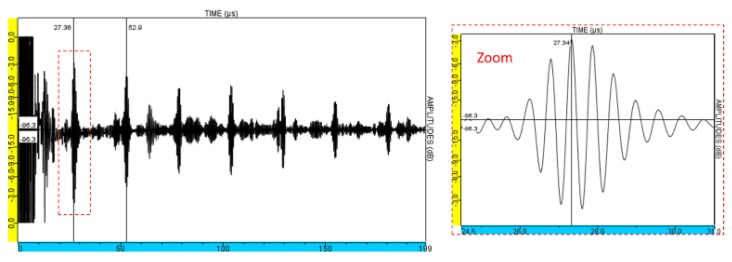
Experimental measurements: A-scan obtained with the EMAT probe in pulse echo mode. No focusing, aluminum block of 80 mm.

The L-wave backwall echo of the block described in Section 4.1 and received when no delay law was applied in either emission or reception was measured. The A-scan obtained is presented in Figure 14.

Considering the velocity of the longitudinal ultrasonic waves in aluminum, approximatively 6300 m/s, and the block thickness, 80 mm, the echo observed at 27 µs is the first backwall echo. The second backwall echo is observed at 52.9 µs. Several echoes of weaker amplitude that appear between the multiple backwall echoes can be noticed. 

**Figure 15:**
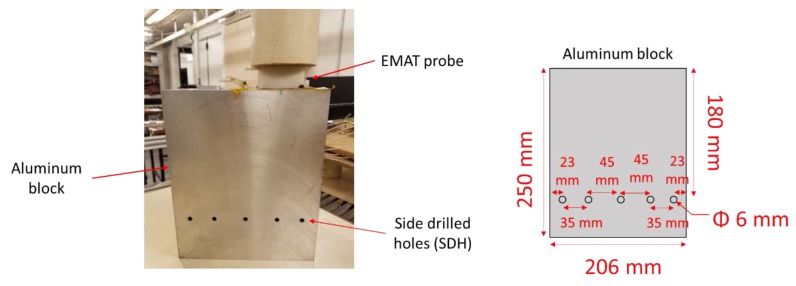
Experimental setup and geometric characteristics of the aluminum block used for the echo measurements on side-drilled holes.

Then, the echoes of five Side-Drilled Holes (SDH) 6 mm in diameter located at 180 mm depth in a 250-mm-thick aluminum block were measured. The characteristics of this block and the experimental setup are shown in Figure 15.

**Figure 16:**
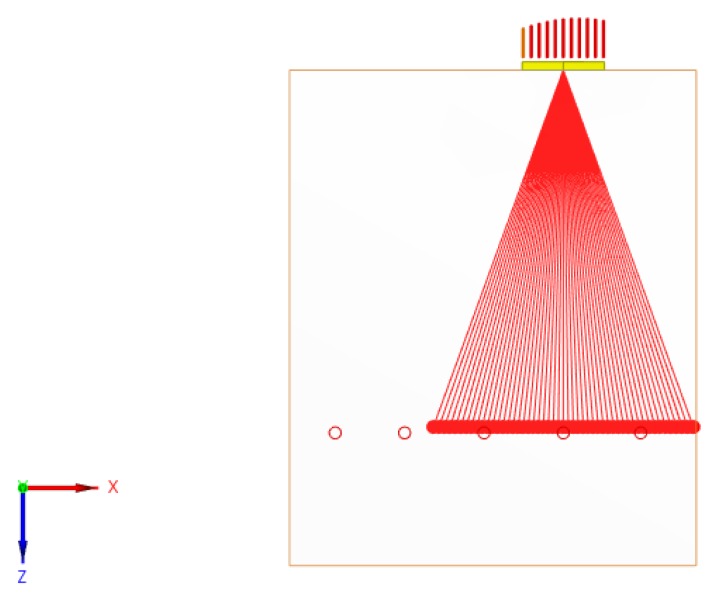
Representation of the sectorial scanning configuration for the inspection of the side-drilled holes.

Fifty delay laws were applied in order to realize an angular deflection of the L-wave beam (sectorial scanning) between -20° and +20° with a focusing at 180 mm. This distance corresponds to the depth of the SDH. It is greater than the near-field distance of the EMAT, but this block was the only aluminum block with SDH available for this study. This sectorial scanning configuration is presented in Figure 16.

**Figure 17:**
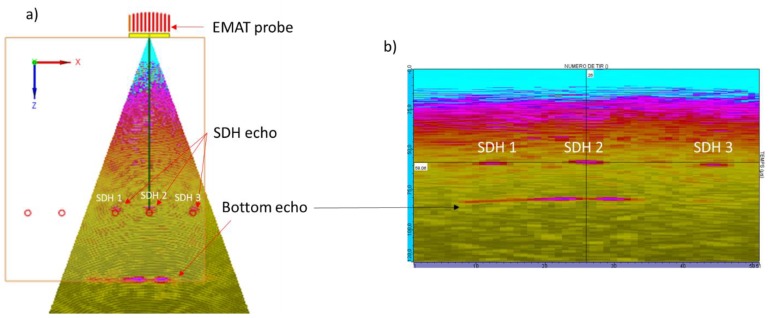
Experimental results. S-scans obtained on the aluminum block (250 mm thickness) with 3 SDHs (6 mm diameter) at 180 mm depth.

In Figure 17, the measured S-scan (Sectorial scan) is represented: in the figure a), the S-scan is reconstructed in the block using the L-waves time of flight and on the figure b) the S-scan is displayed without reconstruction with the fifty laws applied on the x-axis and the time on the y-axis.

**Figure 18:**
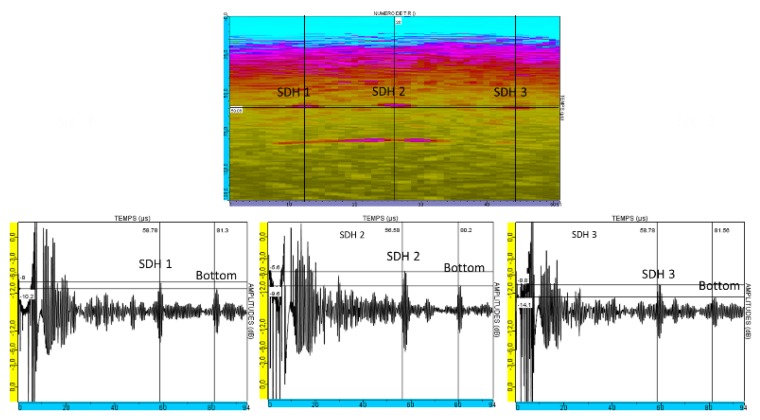
Experimental results. B-scans and A-scans of the 3 SDHs: A high pass filter was applied to eliminate low-frequency noise.

The A-scans extracted from the S-scan at the maximum amplitude of each SDH’s echo are presented in Figure 18.

It may be observed that the dead zone spreads at over 40 µs. Beyond this zone, the 3 SDH responses were detected, as well as the response of the backwall of the block. In each A-san, both echoes are marked with a vertical cursor. 

As expected, the echo of the SDH 2 (identification on Figure 17 and Figure 18) is detected with the best SNR (Signal to Noise Ratio), as it is obtained using the non-deflected beam, whereas the SDH 1 and SDH 3 are detected, with deflections of 11.5° and 15.2°, respectively. 

**Figure 19:**
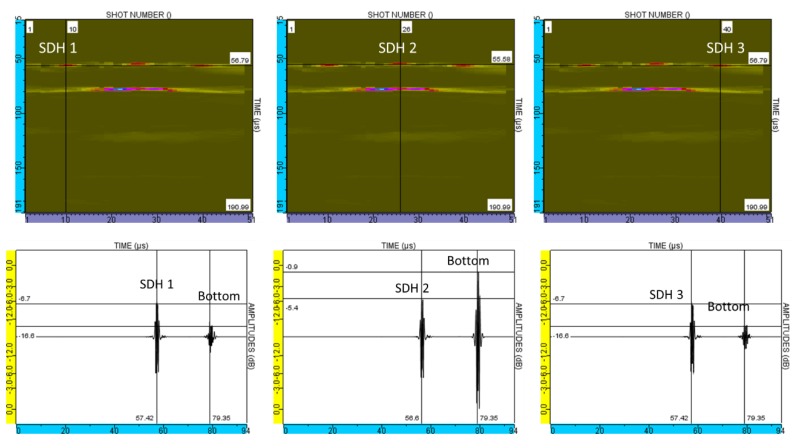
Simulation results with CIVA software. B-scans and A-scans of the 3 SDHs in the 250 mm aluminum block described in.

Between the SDHs and the backwall echoes, other echoes were observed that are not reproduced in the simulation of this experimental test (Figure 19, note that CIVA does not simulate the echoes observed in the dead zone). They were not related to transversal waves that might appear in the aluminum blocks as, if so, these echoes would be present in the simulated images when CIVA computes the T-wave contributions. 

The experimental measurements and simulation results correspond: the SDH echoes and the backwall echoes are detected during the same range of time. However, it can be noticed that the dead zone is not taken into account in the CIVA simulation.

Under laboratory conditions, the results obtained on aluminum blocks with this new EMAT were improved compared to the results obtained with the previous EMAT (higher SNR and better deflection of the ultrasonic beam). 

## 6. Conclusions

Phased array EMAT sensors offer a significant potential for use in liquid sodium, as illustrated with simulated and experimental results obtained with an EMAT prototype manufactured at the French CEA Non-Destructive Testing Department. The prototype’s ability to steer and focus the ultrasonic beam to the desired focal spots using electronic delay laws was demonstrated. The low sensitivity of the EMAT described in this paper is still a drawback and a limitation to its application scope, despite the method proposed to improve it. However, laboratory tests showed a sensitivity high enough to image SDHs in aluminum blocks. Moreover, a comparison between the results obtained under laboratory conditions with the previous EMAT version (which provides some results in liquid sodium) and the new one shows the sensitivity improvement of the latter. Thus, good results in liquid sodium environments can be expected. The next step of this study is to perform tests under a liquid sodium environment at the Cadarache center of CEA by the end of 2019.
